# The decreased serum levels of interleukin-38 in patients with gout and its clinical significance

**DOI:** 10.3389/fimmu.2024.1434738

**Published:** 2024-10-17

**Authors:** Hua Huang, Yinxin Zhou, Yan Li, Hui Zhao, Xiudi Wu, Mingcai Li

**Affiliations:** ^1^ Department of Rheumatology and Immunology, The First Affiliated Hospital of Ningbo University, Ningbo, China; ^2^ School of Basic Medical Sciences, Health Science Center, Ningbo University, Ningbo, China; ^3^ Department of Clinical Laboratory, Ningbo No.6 Hospital Affiliated to Ningbo University, Ningbo, China

**Keywords:** gout, interleukin-38, uric acid, inflammation, biomarker

## Abstract

**Background:**

Interleukin (IL)-38 is a newly discovered anti-inflammatory cytokine. However, its concentration and clinical significance in patients with gout remain unclear. This study aimed to investigate the levels of IL-38 in patients with gout and evaluate their clinical significance.

**Methods:**

Thirty-two patients with active gout, 27 patients with inactive gout, and 20 negative controls (NCs) were included in the study. Clinical parameters, including white blood cell count, C-reactive protein, serum amyloid A, erythrocyte sedimentation rate, uric acid, urea, creatinine, alanine aminotransferase, aspartate aminotransferase, glutamyl transpeptidase, and glycoserated serum protein, were obtained from laboratory tests of blood samples. The serum concentration of IL-38 was determined using enzyme-linked immunosorbent assay. Spearman’s correlation analysis and receiver operating characteristic curve assessments were used to investigate the role and diagnostic value of IL-38 in gout.

**Results:**

Patients with active and inactive gout exhibited significantly lower serum IL-38 levels than NCs. No significant differences were observed between the two gout groups. A negative correlation was observed between IL-38 and white blood cell counts, whereas a positive correlation was found between IL-38 and creatinine levels. Furthermore, IL-38, either alone or in combination with uric acid, demonstrated substantial diagnostic potential.

**Conclusion:**

The findings suggest that the decreased serum levels of IL-38 in patients with gout compared to that in NCs indicates that IL-38 may have immunomodulatory effects on gout inflammation and possesses clinical application value.

## 1 Introduction

Gout is a common type of joint inflammation caused by abnormal purine metabolism (1, 2). The overall prevalence of gout in China is approximately 1% and continues to increase annually (3). Gout mainly affects men, and the incidence of gout in women does not increase significantly until menopause (2). Acute episodes of gout are often characterized by severe pain, redness, and limited joint movement that lasts from several hours to tens of hours (4, 5). The first acute episode in a person with gout is usually self-limiting, occurring within a week or two when the signs and symptoms of arthritis completely subsided (4). However, this reprieve is temporary, as without proper treatment, the arthritic symptoms of gout can recur, worsen, and even turn into chronic gout (4, 6).

Accumulation of monosodium urate (MSU) crystals in patients with gout stimulates an inflammatory response via two pathways. First, toll-like receptor 2 and 4 synthesize pro-interleukin (IL)-1β and inflammatory components in macrophages or monocytes via the nuclear transcription factor-κB signaling pathway. Subsequently, the MSU crystals stimulate the assembly of the inflammasome NOD-like receptor thermal protein domain associated protein 3 (NLRP3), activating the cysteinyl aspartate-specific proteinase-1, which cleaves pro-IL-1β into IL-1β. The inflammasome mediates the binding of IL-1β to IL-1β receptors, inducing acute inflammatory responses (2, 7). Uric acid-lowering therapy is a comprehensive strategy for the long-term management of gout. During acute flares, colchicine, non-steroidal anti-inflammatory drugs, and corticosteroids, are commonly administered in clinical treatment (4, 5). Furthermore, due to the vital role of IL-1 inflammation in gout pathogenesis (8), IL-1 inhibitors are also utilized in clinical treatment (7, 9).

First discovered in 2001, IL-38 is a novel cytokine belonging to the IL-1 family (IL-1F), also known as IL-1F10 (10). IL-38, originating from B-lymphocytes, peripheral blood mononuclear cells, and various immune cells, is expressed in multiple tissues, such as the skin, tonsils, spleen, and thymus (11, 12). IL-38 shares amino acid homology with IL-1 receptor antagonists (IL-1Ra) and IL-36Ra (13). In addition, IL-38 can bind to specific receptors, such as IL-36 receptor (IL-36R), IL-1R1, and IL-1 receptor accessory protein-like 1 (IL-1RAPL1), to exert its biological functions (12, 14). IL-38 regulates autoimmune diseases and exhibits anti-inflammatory effects in the inflammatory response (12, 15). The IL-36R pathway has been extensively studied, and its involvement in autoimmune diseases and inflammatory conditions, such as systemic lupus erythematosus (16), rheumatoid arthritis (17), ankylosing spondylitis (18), and asthma (19), has been reported. The presence of both the full-length and truncated forms of the recombinant IL-38 protein (20) is controversial regarding the activation of the IL-1R1 pathway (21). The IL-1RAPL1 pathway is highly associated with immune and tumor processes (21). The full-length and truncated recombinant IL-38 proteins can bind to IL-1RAPL1 to promote or inhibit inflammation, respectively (20). *IL-38* gene overexpression can modulate the balance between IL-1β and IL-1R, thereby controlling the inflammatory response (22). In addition, a cardiovascular study highlighted that IL-38 inhibits the activation of the NLRP3 inflammasome, inhibiting inflammation (23).

In summary, a potential association between IL-38 and gout exist, indicating that IL-38 controls the occurrence and development of gout by binding to specific receptors and inhibiting the inflammatory response. Therefore, we hypothesized that IL-38 can be a novel biomarker for gout and provide valuable insights into the diagnosis and treatment of this disease. This study aimed to investigate the effect of IL-38 on gout by measuring the serum levels of IL-38 in patients with active and inactive gout.

## 2 Materials and methods

### 2.1 Participants

**Figure 1:**
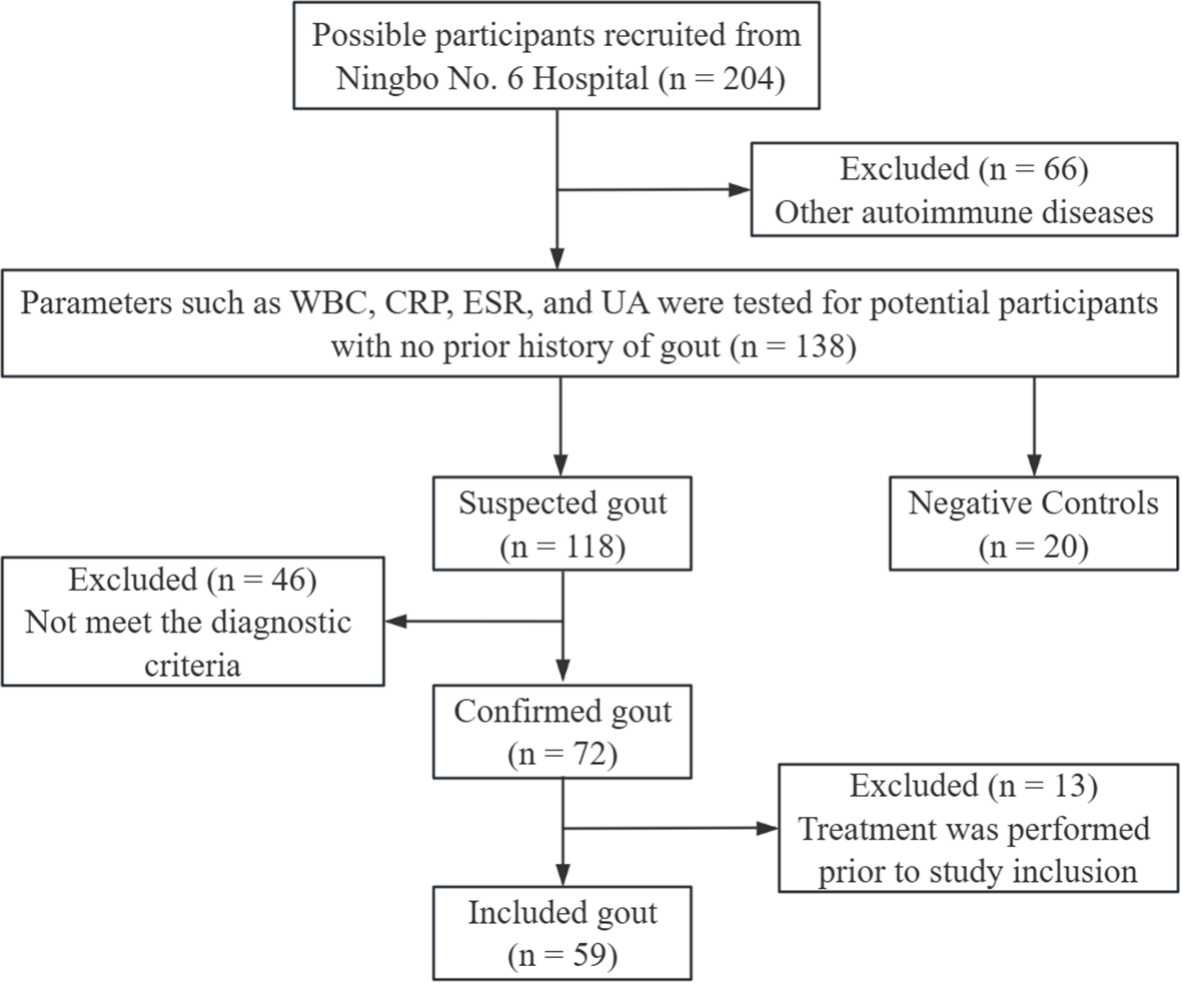
Participants screening process. WBC, white blood cell count; CRP, C-reactive protein; ESR, erythrocyte sedimentation rate; UA, uric acid.

Fifty-nine patients with gout and 20 negative controls (NCs) from the Ningbo No. 6 Hospital from June 2022 to November 2022, were included in the study. The detailed screening process is illustrated in **Figure 1**.

Due to the high prevalence of gout in men, male gout patients older than 18 years were included. The diagnosis of gout is confirmed by two or more rheumatologists using diagnostic criteria (24). On the other hand, hormone administration, infection, tumor, autoimmune disease, and other diseases were excluded, as well as gout related treatment prior to admission. Active and inactive gout were distinguished in 32 and 27 patients, respectively, depending on their gout episode and subjective feelings (25, 26). NCs were obtained from outpatient patients. Their inclusion and exclusion criteria are as follows. (1) No obvious signs of disease were found during routine examination; (2) Age over 18 years old; (3) Male; (4) Exclude hormone administration, infections, tumors, autoimmune diseases, and other diseases especially gout.

This study was approved by the Ethical Review Board of the relevant institutions and conducted in accordance with the Declaration of Helsinki. Informed consent was obtained from all participants.

### 2.2 Clinical data

The age and sex of all participants were recorded prior to inclusion in the study. Clinical data, including white blood cell (WBC) count, C-reactive protein (CRP), serum amyloid A (SAA), erythrocyte sedimentation rate (ESR), uric acid (UA), urea (UR), creatinine (CR), alanine aminotransferase (ALT), aspartate aminotransferase (AST), glutamyl transpeptidase (GGT), and glycoserated serum protein (GSP), total cholesterol (TC), and triglyceride (TG), were obtained from laboratory tests of blood samples. Blood samples were collected from the participants after fasting for 12 h. Blood samples were kept at room temperature (20–25°C) for 2 h. Subsequently, the samples were centrifuged at 1,000 *g* for 15 min at 2-8°C to separate the serum. Laboratory tests were performed using automated blood (BC-6800, Mindray) and biochemical (AU640, Olympus Corporation) analyzers.

### 2.3 Quantification of serum IL-38

The IL-38 concentration in the serum samples of the participants was determined using enzyme-linked immunosorbent assay (ELISA) kit (CSB-EL011615HU, Cusabio Technology, Wuhan, China) according to the manufacturer’s instructions. The precision within an assay and between assays of the ELISA kits was less than 8% and 10%, respectively. The assay detection range was 31.25–2,000 pg/mL.

The experimental procedure is summarized as follows: melted serum and diluted standards were added to an enzymatic plate balanced at room temperature for 30 min. After incubating at 37 °C for 2 h, the supernatant was removed, and the biotin antibody and horseradish peroxidase avidin were added and incubated at 37°C for 1 h. Finally, after adding 3,3´,5,5´-tetramethylbenzidine (TMB) solution for 30 min in the dark, the reaction was ended using termination solution. Then, the absorbance was measured at 450 nm using a spectrophotometer (Multiskan GO; Thermo Fisher Scientific). A standard curve was constructed using equally diluted standard solutions.

### 2.4 Statistical analysis

All statistical analyses in this study were performed using GraphPad Prism 9.0 version. The measurement data were tested using the Shapiro–Wilk test for normal distribution. According to the distribution, the measurement data were presented as mean ± standard deviation (SD) or median and interquartile range (IQR). For the analysis of the three sets of data, one-way analysis of variance (ANOVA) or the Kruskal–Wallis test was used. In multiple comparisons between the three groups, the Dunn’s *post-hoc* test was used. Correlations between clinical data were analyzed using rank correlations. The diagnostic value of the disease was demonstrated using receiver operating characteristic (ROC) curves. All tests were two-sided, with a test level of 0.05.

## 3 Results

### 3.1 Characteristics of participants

The ages of the participants in the three groups were comparable (*P* = 0.195). The levels of UR (P = 0.345) and GSP (*P* = 0.088) in serum did not differ among the participants. The pairwise comparison using Dunn’s *post-hoc* test showed that all other indicators of patients with active gout were different from those of NCs. However, only SAA, UA, CR, and ALT levels were significantly different in patients with inactive gout. Detailed clinical data are presented in **Table 1**.

**Table 1:** Characteristics of participants.

Variables	Active gout	Inactive gout	NCs	*P*-value
N	32	27	20	/
Sex (Male%)	100%	100%	100%	/
Age (y)	42.4 ± 12.8	39.7 ± 13.8	35.7 ± 11.5	0.195
WBC (10^9^/L)	8.7 (7.6, 10.4)^a^	6.7 (5.5, 7.8)	5.7 (5.1, 6.9)	< 0.001
CRP (mg/L)	12.3 (5.6, 31.2)^a^	1.4 (0.6, 2.5)	1.3 (1.2, 1.5)	< 0.001
SAA (mg/L)	55.9 (6.9, 227.8)^a^	5.2 (2.6, 9.4)^b^	2.0 (1.6, 2.8)	< 0.001
ESR (mm/h)	26.5 (11.0, 39.3)^a^	8.0 (4.0, 16.0)	8.0 (4.2, 10.75)	< 0.001
UA (μmol/L)	445.5 (359.5, 566.5)^a^	442.0 (376.0, 580.0)^b^	345.5 (319.8, 378.5)	< 0.001
UR (mmol/L)	4.1 (3.6, 5.3)	4.7 (4.1, 5.6)	4.9 (3.6, 5.7)	0.345
CR (μmol/L)	85.4 (75.7, 91.8)^a^	82.9 (77.3, 86.9)^b^	72.5 (65.2, 78.7)	0.001
ALT (U/L)	40.5 (27.0, 64.8)^a^	38.0 (28.0, 54.0)^b^	25.5 (20.3, 30.8)	0.002
AST (U/L)	24.0 (19.0, 38.0)^a^	24.0 (20.0, 32.0)	19.5 (17.0, 23.0)	0.037
GGT (U/L)	43.5 (29.8, 92.8)^a^	33.0 (23.0, 72.0)	23.0 (16.5, 33.3)	0.007
GSP (mmol/L)	2.0 ± 0.3	1.9 ± 0.3	2.0 ± 0.2	0.088
TC (mmol/L)	5.0 (4.4, 5.4)	5.1 (4.4, 5.9)^b^	4.4 (3.9, 5.1)	0.031
TG (mmol/L)	2.0 (1.4, 2.5)a	2.7 (1.8, 3.7)^b^	1.1 (0.7, 1.4)	< 0.001

NCs, negative controls; WBC, white blood cell count; CRP, C-reactive protein; SAA, serum amyloid A; ESR, erythrocyte sedimentation rate; UA, uric acid; UR, urea; CR, creatinine; ALT, alanine aminotransferase; AST, aspartate aminotransferase; GGT, glutamyl transpeptidase; GSP, glycoserated serum protein; TC, total cholesterol; TG, triglyceride. Multiple group comparisons of age were made using one-way analysis of variance. Multiple group comparisons of other indicators were made using Kruskal-Wallis test. Pair-to-pair comparisons between multiple groups were made using Dunn’s *post-hoc* test. The P-value was obtained by one-way analysis of variance, Kruskal-Wallis test and the Dunn’s *Mpost-hoc* test. ^a^ Active gout compared with NCs, *P* < 0.05. ^b^ Inactive gout compared with NCs, *P* < 0.05.

### 3.2 Serum IL-38 concentrations

**Figure 2:**
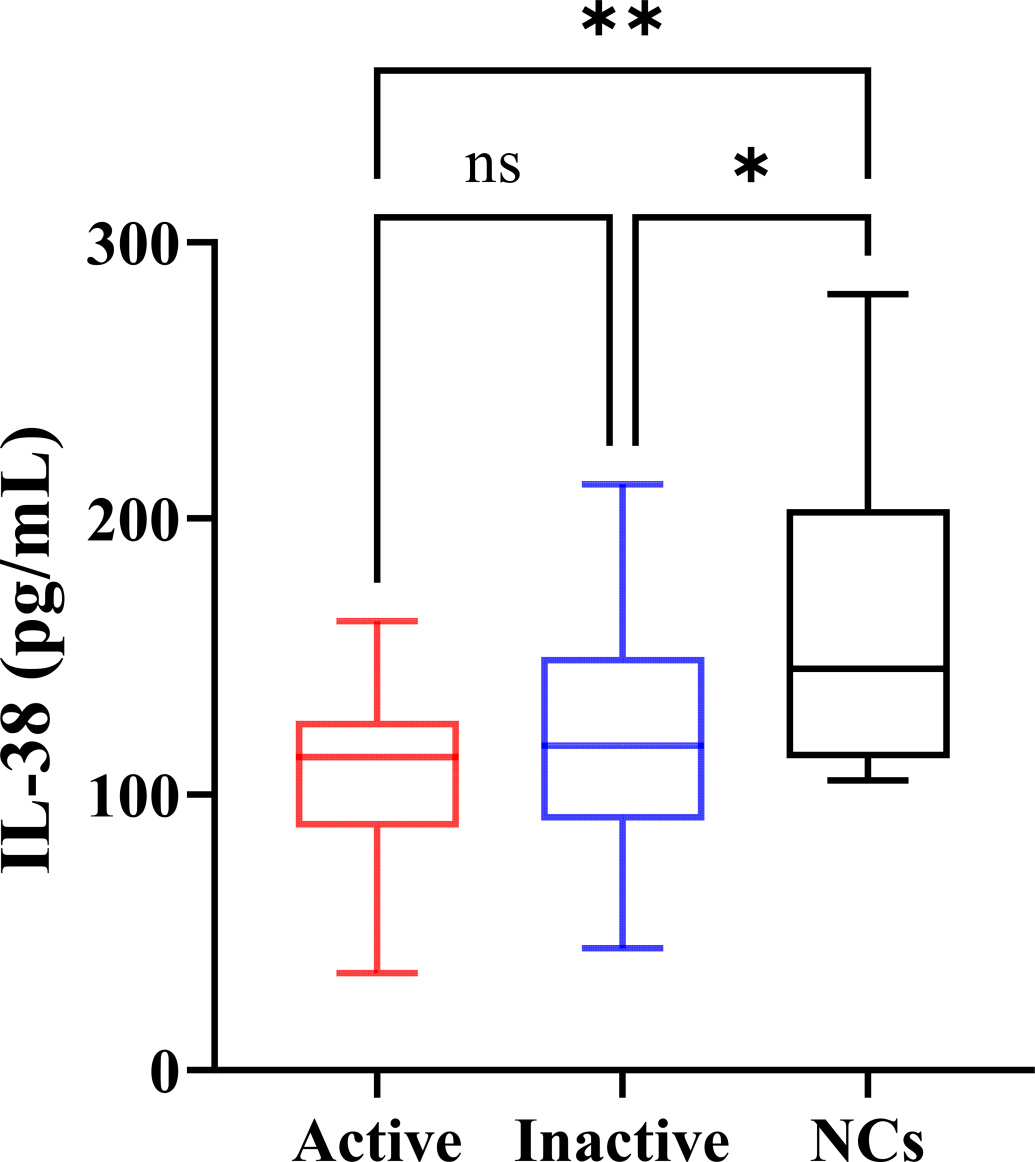
Serum IL-38 concentrations of the participants. NCs, negative controls. The *P*-values of the three groups were obtained by Kruskal-Wallis test. The *P*-value of pairwise comparison was obtained by Dunn’s *post-hoc* test. ^*^ *P* < 0.05, *^*^ *P* < 0.01, ns, no significance.

Serum IL-38 concentrations of the participants are shown in **Figure 2**. The levels of IL-38 in active gout, inactive gout, and NCs were 113.45 (88.04, 126.75), 117.55 (90.48, 149.89), and 145.51 (113.12, 203.37) pg/mL, respectively. The nonparametric test showed that IL-38 concentrations in the three groups differed. The Dunn’s *post-hoc* test showed that both active gout patients (*P* = 0.0013) and inactive gout patients (*P* = 0.0356) had lower IL-38 levels than NCs. However, no significant difference between the two groups was observed (*P* = 0.9667).

### 3.3 Correlation analysis

**Figure 3:**
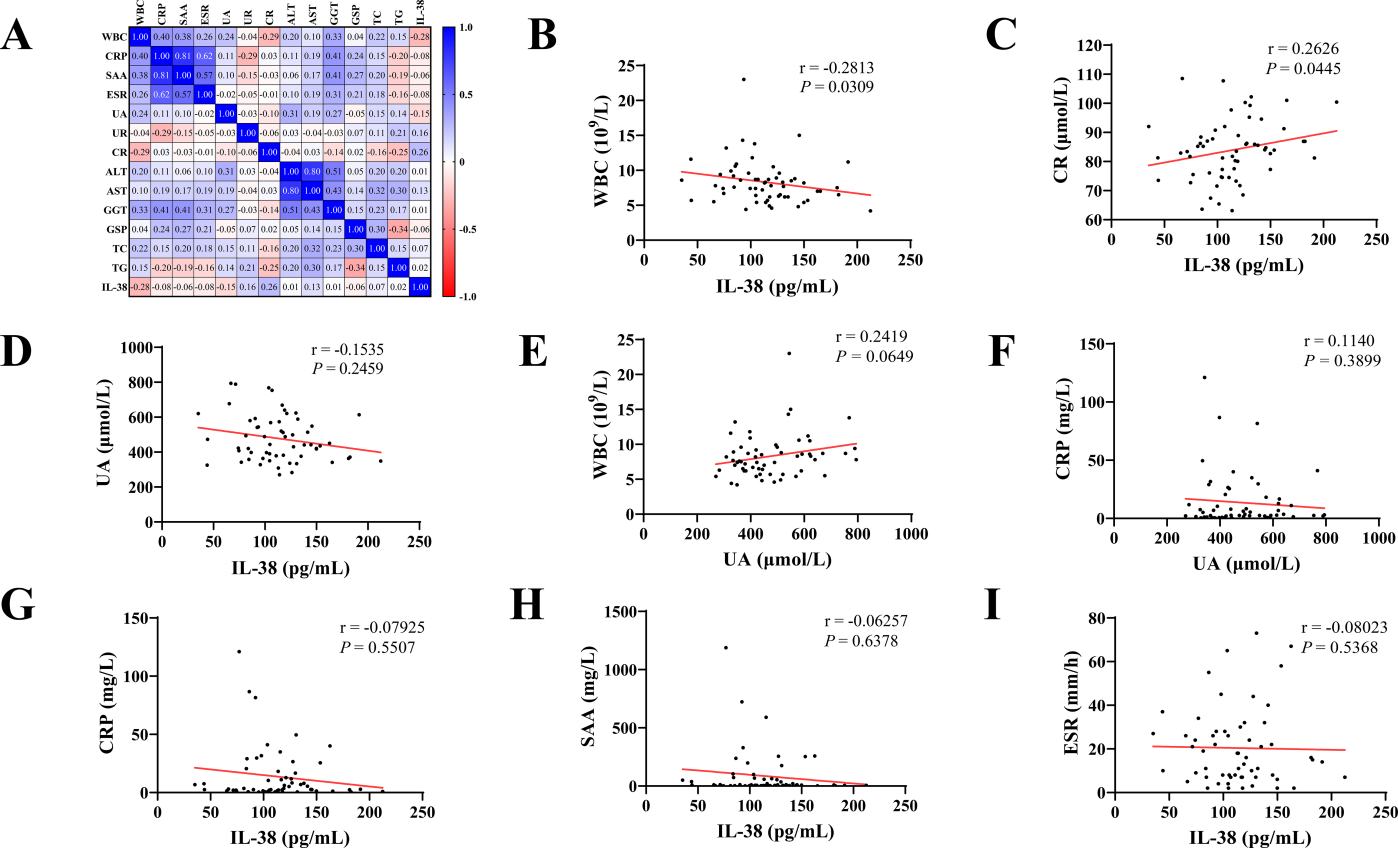
Correlation analysis of clinical indicators. **(A)** Heat map with Spearman correlation analysis of all indicators in gout patients. **(B–I)** Correlation analysis between various clinical indicators. WBC, white blood cell count; CRP, C-reactive protein; SAA, serum amyloid A; ESR, erythrocyte sedimentation rate; UA, uric acid; UR, urea; CR, creatinine; ALT, alanine aminotransferase; AST, aspartate aminotransferase; GGT, glutamyl transpeptidase; GSP, glycoserated serum protein; TC, total cholesterol; TG, triglyceride; IL-38, interleukin-38.

The correlation between the clinical data of the patients was demonstrated using Spearman’s correlation analysis (**Figure 3**). In patients with gout, IL-38 levels was correlated with WBC counts (r = -0.2813, *P* = 0.031) and CR levels (r = 0.2626, *P* = 0.045). No correlation was found between IL-38 and UA levels (r = -0.1535, *P* = 0.2459). Similarly, the association between WBC counts (r = 0.2419, *P* = 0.0649) and CRP levels (r = 0.1140, *P* = 0.3899), two common indicators of inflammation, and UA levels was not statistically significant. No correlation was found between IL-38 and CRP (r = -0.07925, *P* = 0.5507), SAA (r = -0.06257, *P* = 0.6378), ESR (r = -0.08203, *P* = 0.5368) and other inflammatory indicators.

### 3.4 ROC curve analysis

**Figure 4:**
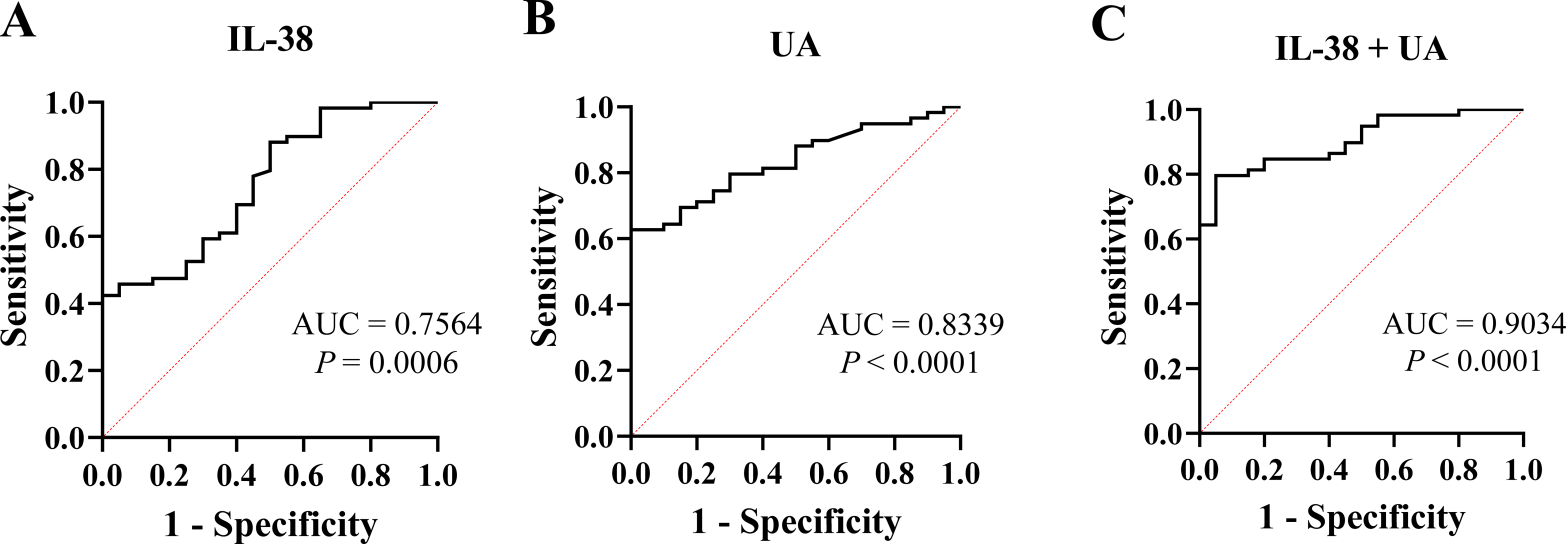
The receiver operating characteristic curve of IL-38 and UA. **(A)** IL-38 was used to diagnose gout. **(B)** UA was used for diagnose gout. **(C)** IL-38 combined with UA were used to diagnose gout. UA, uric acid; IL-38, interleukin-38; AUC, areas under the curve.

The ROC curve for evaluating the diagnostic value of gout showed that the areas under the curve (AUC) for IL-38 and UA levels were 0.7564 and 0.8339, respectively. Combined analysis of these two indicators showed that the AUC increased to 0.9034 (**Figure 4**).

## 4 Discussion

This study was conducted in Ningbo, China, and included 79 participants. Based on the ELISA results, this study is the first to identify differences in serum IL-38 levels between patients with gout and negative individuals. The serum IL-38 levels of patients with gout were lower than that of NCs, and the level of IL-38 in patients with active gout was lowest. Furthermore, correlation analysis demonstrated a negative association between IL-38 and WBC count, whereas a positive correlation was observed between IL-38 and CR. The ROC curve analysis supported the diagnostic value of IL-38.

Although the role of IL-38 in other types of arthritis, such as osteoarthritis and rheumatoid arthritis, has been gradually uncovered (15, 17), its significance in gout remains unclear. Some studies show that hyperuricemia is one of the independent risk factors for gout (1, 3, 27). We found that serum IL-38 levels were lower in patients with hyperuricemia than in healthy individuals (28). Although not all patients with hyperuricemia will eventually develop gout, the findings are valuable for this study. A previous study showed that recombinant IL-38 protein alleviated MSU-induced arthritis in mice (29). In other studies, serum IL-38 levels were found to be lower in patients with gout than in RA, which is also osteoarthritis (30). Unfortunately, studies of gout patients and healthy people or negative controls are not discussed at this time. In this study, patients with gout had lower IL-38 concentrations than NCs. Patients with active gout showed a slightly greater decrease in IL-38 levels than those with inactive gout. However, no statistical difference was observed between the two groups. These results suggest that IL-38 plays a vital role in the inflammatory response of gout. According to previous studies and existing mechanisms, the decreased levels of IL-38 in serum of patients with gout may be because IL-38 inhibits the action of IL-1β to prevent the occurrence and development of the disease (22, 29). In patients with gout, serum IL-38 failed to recover from the low level of the active period to the high level of negative individuals, suggesting that the disappearance of symptoms in patients with an inactive gout period is a temporary self-limitation and a pathogenic basis for repeated acute flares exist.

As a non-specific inflammatory indicator, the WBC count showed a negative correlation with IL-38 levels, indicating that IL-38 is related to the inflammatory response in gout. Studies on the correlation between IL-38 and CR are scarce. A correlation between CR and IL-1β in hyperuricemia has been found. Therefore, it is speculated that CR may be somewhat related to IL-38 as an intermediary variable (31). In this study, no correlation was found between UA and inflammatory markers, such as WBC and CRP, which is consistent with the results of other studies (32, 33). Probably, UA level, a gout-specific indicator, is essentially the result of the metabolic disorder; therefore, UA is not closely associated with inflammation in gout. Consequently, no correlation was expected between IL-38 and UA levels. The ROC curve analysis showed an AUC of 0.7564 for IL-38, indicating its diagnostic value for gout. Although lower than that of UA, the combined diagnostic capability of UA and IL-38 was 0.9034. Moreover, we believe that IL-38 plays a role in the inflammatory response of gout, and whether it is in the active phase has no significant influence on serum IL-38 content. However, the mechanism of IL-38 action in gout has not been fully elucidated in this study.

This pioneering study investigated differences in IL-38 levels between patients with gout and negative individuals. Additionally, we categorized the patients into active and inactive gout phases to explore the changes in serum IL-38 levels at different time points. The possible role and diagnostic value of IL-38 in gout were demonstrated using correlation and ROC curve analyses. This study has some limitations. First, as a single-center case-control study, the admission rate bias was unavoidable. Bias was controlled as much as possible using measures such as matching and objective indicators. However, we do not believe that the existence of bias will make the results unreliable. Second, the small sample size may have resulted in less stable outcomes. In a follow-up study, we will conduct a multicenter, large-sample study and animal experiments to elucidate the specific mechanism of IL-38 in the occurrence and development of gout.

In conclusion, we found, for the first time, that serum IL-38 concentration decreased in patients with gout, suggesting its potential role in gout inflammation.

## Data Availability

The raw data supporting the conclusions of this article will be made available by the authors, without undue reservation.
